# Exploring the validity and internal consistency of the 12-point Mediterranean Diet Adherence Screener among Lebanese pregnant women

**DOI:** 10.1371/journal.pone.0351396

**Published:** 2026-06-23

**Authors:** Rana Rizk, Maroun Khattar, Pascale Salameh, Rana Mahfouz, Yonna Sacre, Maha Hoteit

**Affiliations:** 1 Department of Nutrition and Food Science, School of Arts and Sciences, Lebanese American University, Byblos, Lebanon; 2 Institut National de Santé Publique, d’Épidémiologie Clinique et de Toxicologie-Liban (INSPECT-LB), Beirut, Lebanon; 3 Faculty of Public Health, PHENOL Research Group (Public Health Nutrition Program-Lebanon), Lebanese University, Beirut, Lebanon; 4 Faculty of Pharmacy, Lebanese University, Hadath, Lebanon; 5 Department of Primary Care and Population Health, University of Nicosia Medical School, Nicosia, Cyprus; 6 Department of Nutrition and Food Sciences, Faculty of Arts and Sciences, Holy Spirit University of Kaslik (USEK), Jounieh, Lebanon; Lusofona University of Humanities and Technologies: Universidade Lusofona de Humanidades e Tecnologias, PORTUGAL

## Abstract

Although 14-MEDAS is widely used for dietary assessment, it has not been validated in pregnant Lebanese women. This study assesses its validity and internal consistency against a Food Frequency Questionnaire (FFQ) and 24-hour recalls (24HR). A cross-sectional study with 500 pregnant women was conducted between March and October 2023. Food consumption data were gathered using a validated FFQ and three non-consecutive 24-HR to validate the 14-MEDAS. Adherence thresholds were adjusted for the removal of the alcohol item (contraindicated during pregnancy) and olive oil quantity (low communalities: < 0.30), leading to a maximum score of 12 (12-MEDAS). Internal consistency, as measured by KR-21, was acceptable (0.76) reflecting the formative nature of the scale. Factor analysis revealed a four-factor structure explaining 46.28% of the variance. The 12-MEDAS demonstrated suboptimal relative validity. While it provides a quick assessment of adherence to the Mediterranean Diet, discrepancies between the 12-MEDAS and reference methods (FFQ and 24HR) likely stem from the tool’s inability to account for pregnancy- and locally-specific food groups and habits, as well as measurement errors inherent to self-reported methods. The results warrant a cultural and physiological adaptation, such as adjusting for the recommended alcohol abstinence, and considering local food sources and eating habits.

## Introduction

Maternal nutrition plays a significant role in fetal development; it also influences the immediate and future health outcomes for both the mother and fetus [1,2]. The Mediterranean Diet (MD) is known globally for its health benefits, including the prevention of many noncommunicable diseases such as obesity, diabetes, cardiovascular diseases, and cancer [3,4]. This diet encourages the consumption of fruits, vegetables, nuts, olive oil, pulses, and whole grain cereals; it is characterized by moderate consumption of fish and poultry, low consumption of red meat, e.g., pork, beef, very low or null consumption of processed meats and sweets, and a light-to-moderate wine consumption with meals [4]. During the preconception and pregnancy periods, the MD is positively associated with the maternal and offspring’s health [5]. For instance, maternal diets adhering to the MD were associated with normal infant birthweight [6], a lower risk for allergies in children [7], and a lower risk of preterm delivery and pre-eclampsia [8].

Given the documented importance of maternal diet during gestation, specifically adherence to the MD, assessing and monitoring it in this population group is crucial. To date, dietary intake assessment studies rely on food frequency questionnaires (FFQ) and 24-hour recalls (24HR) as the main data collection tools [9]. Despite the valuable data they provide, these tools are time-consuming and require trained data collectors [10]. Recently, the Mediterranean Diet Adherence Screener (14-MEDAS), was suggested as an easier-to-administer, less time-consuming, and reasonably valid tool for the assessment of MD adherence compared with the other dietary assessment tools [11]. However, several physiological and behavioral shifts unique to pregnancy can significantly alter its performance. Behaviorally, a critical conflict arises from the screener’s scoring of wine consumption: while the original tool awards a point for intake, pregnant women are clinically advised to practice total alcohol abstinence, causing the tool to penalize safe maternal behavior. Dietetically, pregnancy often triggers food aversions and increased physiological demands, such as the heightened need for dairy-derived calcium, which the 14-MEDAS fails to weigh accurately [10]. Furthermore, in the Lebanese context, traditional dishes, while inherently Mediterranean, often involve preparation methods and food habits that could not be fully captured by Western-validated screeners. For instance, the Lebanese *Meza* style of eating, characterized by small portions of many different items, complicates the “servings per day” logic of the MEDAS.

Although a pregnancy-adapted version of the MEDAS, the 17-item preg-MEDAS, has been validated in Spain [10], this tool may not be reproducible in broader contexts because the participants were a highly specific group at high risk of delivering small for gestational age infants, rather than a representative sample of the general population of pregnant women. Furthermore, the study took place in a high-resource setting characterized by uncommonly low rates of obesity and gestational diabetes. These unique demographic and environmental factors propose that the results might differ significantly when applied to more diverse or lower-resource communities [10]. Additionally, the 14-MEDAS remains the most widely utilized dietary screener by local researchers and practitioners. Specifically, many countries have validated the 14-MEDAS as a tool to assess MD adherence in their corresponding populations, including Germany, United States, United Kingdom, and Korea, using either FFQs or food diaries [11]. Each of those studies reported the 14-MEDAS as a reasonably valid tool with an overall modest-to-fair concordance with the reference tool [11,12]. And, while the standard 14-MEDAS has been validated in numerous countries, Spain remains the only setting where preg-MEDAS has been validated among pregnant women.

In Lebanon, the 14-MEDAS is commonly used in research and clinical settings [13]; however, this tool remains unvalidated. Local validation is essential because a tool’s accuracy is context-dependent: a screener validated for general populations in Europe or the United States may not accurately capture dietary adherence in Lebanese pregnant women due to marked dietary, cultural, and physiological differences. This warrant determining the local validity and internal consistency for our target population of pregnant women. As such, this study was conducted to explore the validity and internal consistency of the 14-MEDAS among a sample of Lebanese pregnant women.

## Materials & methods

### Study design

A cross-sectional survey was conducted between March 1^st^ and October 31^st^, 2023 involving a sample of adult Lebanese pregnant women (N = 500).

### Sample size calculation and sampling technique

In compliance to a 2018–2019 report done by the Lebanese Republic’s Central Administration of Statistics regarding the number of women of reproductive age (18–49 years), the population’s size was N = 1,244,000 [14]. As such, the sample size was determined using the following formula [15], with 95% confidence interval: 𝐧 = (𝐭2𝐍)/(𝐭2 + (2𝐞)2(𝐍−1)). In this formula, the sample size is represented by “n”, while “t” refers to the margin coefficient subtracted from the confidence level (1.96), “N” refers to the initial population’s size, and “e” refers to a 5% margin of error.

Based on the above, a total of 384 pregnant women were required, and we considered rounding the sample to 500 participants. The reason for oversampling was to increase the estimates’ precision and to account for the nonresponse rate. A convenient sampling approach was employed, with participants recruited from various healthcare settings, including private OBGYN clinics and public primary healthcare centers across all Lebanese governorates. Recruitment was conducted by trained dietitians who approached pregnant women during their scheduled prenatal visits. Eligible participants were provided a detailed briefing on the study’s objectives and methods before providing informed consent. Overall, we approached 600 pregnant women among which 70 did not respond, leading to an 11.7% non-response rate. In the initial phases of the research, gynecologists were supportive and helped facilitate contact with pregnant women: they informed their clients about the objectives of the study and welcomed trained dietitians to invite their clients to participate in the study. The participants were distributed from all governorates with varying proportions from each governorate.

### Ethical considerations

The Research Ethics Committee (REC) of the Higher Center for Research (HCR) at the Holy Spirit University of Kaslik (USEK) approved the study protocol on 13/01/2023. Data were collected in accordance with the Declaration of Helsinki. Participants provided written informed consent after being briefed on the study’s purpose and methods, ensuring a voluntary and transparent process. To uphold the “no-harm” principle, data collection was performed in private settings to guarantee participant confidentiality. All responses remained anonymous, with physical records stored securely and digital data maintained on password-protected devices for strictly academic use.

### Study population

Participants who met the following criteria were eligible: Lebanese citizens living in Lebanon, aged 18–49 years old, currently pregnant at various stages of pregnancy, did not have any chronic diseases, did not develop gestational diabetes or pre-eclampsia during pregnancy. While lactating women were generally excluded, those nursing an older child while pregnant were eligible to participate.

### Data collection

This study utilized a retrospective secondary analysis of dietary data collected in the six months before conducting the current analysis. The survey included all study tools for data collection, required forty-five minutes to be completed, and was conducted face-to-face by trained dietitians after determining participants’ eligibility, recruiting them, and providing ample information about the study’s objectives and procedures. Whenever needed, study tools were translated from English to Arabic, the native language of the participants. Before the survey’s administration, the Arabic version was pilot tested for clarity, context specificity, and validity. This also allowed us to assess the time needed to fill the survey, and to identify and exclude issues with data gathering and entry. Thirty pregnant women not included in the current study contributed to the pilot testing. After the pilot testing, the tool was judged as clear and applicable, with minor changes applied to the wording. The survey included the following sections:

#### Sociodemographic, personal, and health-related information.

Recorded information included age, residency, marital status, household size, and number of rooms, participants’ educational level, employment status, household income, number of children, and status of pregnancy. Household size and number of rooms were collected to calculate the crowding index; the latter was considered as a proxy indicator of the household’s socioeconomic status [16].

#### Food consumption.

A 157-item validated FFQ [17] was used to assess the frequency of food consumed by the study participants using four reference period categories: daily, weekly, monthly, and never. This FFQ showed good relative validity and reproducibility in comparison with three 24-HR involving a heterogeneous sample of adults residing in Lebanon [17]. The categories were then unified into daily intake for every participant (grams per day). Additionally, three non-consecutive days (Monday, Thursday, and Sunday) were used to collect 24HR. Dietitians were well-trained to collect the 24HR. They asked participants to recall all the foods and beverages they ate in the last 24 hours. To help participants remember and more accurately estimate the portion sizes, visual aids and food models were offered when completing the FFQ and 24HR.

#### The 14-MEDAS scale.

The 14-MEDAS used in this study is based on the original PREDIMED protocol [18]. The unmodified tool was administered as it is currently utilized as such in research. The questionnaire was administered in Arabic, using a previous translation used in Lebanese research [13]. Hence, a formal, multi-step cross-cultural adaptation process, including forward and backward translation, expert committee review, and cognitive debriefing, was not conducted for this study.

The 14-MEDAS is based on 14 questions; its total score ranges from 0 to 14, with varying adherence categories distributed as follows: a score ≤5 indicates weak adherence, a score from 6 to 9 indicates moderate to fair adherence, and a score ≥10 indicates good or very good adherence. The 14-MEDAS scores were not obtained through a standalone administration of the screener; instead, they were derived post-hoc from the detailed data collected using a 157-item FFQ and three 24-HR. In detail, food consumption data from the FFQ and 24HR were harmonized with MEDAS criteria by converting reported intake frequencies and portion sizes into the standard units required by the index, such as servings/day or servings/week. For example, monthly or weekly FFQ frequencies were recalculated into daily equivalents to determine if they met the MEDAS cut-offs, allowing for a standardized comparison of adherence to specific MD items across the sample. For both instruments, the same binary scoring thresholds were applied. Each criterion met was assigned a score of 1, e.g., 1 point was awarded for a consumption of ≥2 vegetable servings/day, while failure to meet a criterion resulted in a score of 0. This approach allowed for a direct comparison of the screener’s performance based on recorded dietary intake, ensuring that all three assessment methods referred to the exact same consumption period. A detailed mapping of specific FFQ questions and 24HR food categories is provided in S1 Table.

### Statistical analysis

Data were analyzed using SPSS version 28.0. Distribution normality was assessed via Kolmogorov-Smirnov and Shapiro-Wilk tests and Q-Q plots; due to non-normal distributions, non-parametric tests were employed.

Since wine is originally contraindicated during pregnancy, the question related to wine consumption was excluded, resulting in a 13-item scale. Construct validity of the tool was assessed through Multiple Correspondence Analysis and Exploratory Factor Analysis (EFA). The EFA utilized Principal Component Analysis with Promax rotation, retaining factors with eigenvalues > 1. Sampling adequacy was confirmed via inter-item correlations, anti-image matrices, a Kaiser-Meyer-Olkin (KMO) score > 0.50, and a significant Bartlett’s test of sphericity [19]. Since the retention threshold was set at factor loadings > 0.40 and communalities > 0.30, some items had to be removed: the “how many tablespoons of olive oil do you consume per day?” question referred to as “olive oil quantity” item was excluded due to a low communality (< 0.20), whereas “olive oil use” and “legumes” items were purposefully retained despite marginal values to preserve the conceptual integrity of the MD construct. Accordingly, following the exclusion of the “wine consumption” and “olive oil quantity” items, the total score ranged from 0 to 12, and the tool’s name was modified to 12-MEDAS to reflect the 12-item scale. To maintain consistency with the original PREDIMED classification and avoid systematic misclassification, the adherence thresholds were adjusted proportionally to the 12-item scale, and were categorized based on tertiles, whereby a score of ≤ 5 denoted a weak adherence, a score of 6 denoted moderate to fair adherence, and a score of ≥ 7 denoted good to a very good adherence. All comparative analyses with the FFQ and 24HR were based on the adjusted 12-item scoring system.

Internal consistency of the 12-MEDAS was measured using Cronbach’s alpha and Kuder-Richardson Formula 21 (KR-21) [20]; however, the latter is more appropriate than the former for the scale’s dichotomous scoring, and was preferably adopted. Consequently, while Cronbach’s alpha was calculated for completeness, it is acknowledged as an inherently limited measure for this type of dietary screener [11]. In addition, MEDAS is a formative index rather than a reflective scale. In formative indices, the individual items, i.e., specific food groups, combine to define the underlying concept, i.e., MD adherence, and are not necessarily expected to be highly correlated with one another. Convergent validity (known-groups) with the FFQ and 24HR was evaluated using Kappa agreement coefficients [20–22]. Finally, bivariate associations were evaluated using Spearman’s rank correlation for continuous variables and Goodman and Kruskal’s gamma for ordinal categories. A p-value <0.05 was considered significant.

## Results

### Characteristics of the study participants

**Table 1** shows the sociodemographic and pregnancy-related characteristics of the study participants. About two-thirds of the participants (65.8%) were aged 30 years or below. The majority resided in Beirut and Mount Lebanon (49.2%). Almost all the participants (98%) lived in non-crowded households, and about half (51.8%) were in their second trimester (51.8%).

**Table 1:** Sociodemographic and medical characteristics of study participants (n = 500).

*Sociodemographic Characteristics*		N	%
Age Category (years)	18-30	329	65.8%
	31-49	171	34.2%
Current Residency	Beirut & Mount Lebanon	246	49.2%
	North Lebanon & Akkar	100	20%
	South Lebanon & Nabatiyeh Beqaa & Baalbek-Hermel	98 56	19.6% 11.2%
Crowding Index	Not crowded (≤1 person per room)	490	98%
	Crowded (>1 person per room)	10	2%
Mother Educational Level	Illiterate or school	174	34.8%
	University	326	65.2%
Employment Status	Unemployed Employed	208 292	41.6% 58.4%
Monthly Household Income	<USD 300	295	59%
	≥USD 300	205	41%
Number of Children	0	226	45.2%
	1-3	266	53.2%
	> 3	8	1.6%
***Medical Characteristics***		**N**	**%**
Pregnancy trimester	First trimester	100	20%
	Second trimester	259	51.8%
	Third trimester	141	28.2%

### Factor analysis of 12-MEDAS items

**Table 2** shows the results of the KMO test and Bartlett’s test of sphericity. The KMO measure (0.583) was greater than 0.5, and Bartlett’s test was less than 0.05, indicating that the variables can be used in factor analysis and that the sample is adequate for this type of analysis.

**Table 2:** KMO and Bartlett’s test.

Kaiser-Meyer-Olkin Measure of Sampling Adequacy		0.583
Bartlett’s Test of Sphericity	Approx. Chi-Square	304.490
	Df	66
	Sig.	<0.001

Factor analysis results are shown in **Table 3**. After removing the alcohol consumption question due to its conceptual inadequacy in a population with expected absence of consumption, and the “olive oil quantity” item due to its extremely low communality, the remaining 12 items loaded on four factors, which explained 46.28% of the variation in the data.

**Table 3:** Factor analysis of the 12-MEDAS scale items.

Communalities				
		Initial		Extraction
Do you use olive oil as the main culinary fat?		1.000		0.263
How many vegetable servings do you consume per day		1.000		0.515
How many fruit units (including natural fruit juices) do you consume per day?		1.000		0.400
How many servings of red meat, hamburger, or meat products (ham, sausage...) do you consume per day?		1.000		0.581
How many servings of butter, margarine, or cream do you consume per day?		1.000		0.546
How many sweet or carbonated beverages do you drink per day?		1.000		0.480
How many servings of legumes do you consume per week?		1.000		0.273
How many servings of fish or shellfish do you consume per week?		1.000		0.510
How many times per week do you consume commercial sweets or pastries (not homemade)		1.000		0.602
How many servings of nuts (including peanuts) do you consume per week?		1.000		0.579
Do you preferentially consume chicken, turkey, or rabbit meat instead of veal, pork, hamburger, or sausage?		1.000		0.369
How many times per week do you consume vegetables, pasta, rice, or other dishes seasoned with sofrito		1.000		0.435
Extraction Method: Principal Component Analysis; **Total variance explained = 46.28%**				
**Pattern Matrix after Promax Rotation**				
	**Component**			
	1	2	3	4
How many servings of butter, margarine, or cream do you consume per day?	0.730			
How many servings of red meat, hamburger, or meat products (ham, sausage, etc.) do you consume per day?	0.711			
How many sweet or carbonated beverages do you drink per day?	0.644			
How many servings of nuts (including peanuts) do you consume per week?		0.636		
How many fruit units (including natural fruit juices) do you consume per day?		0.571		
Do you use olive oil as the main culinary fat?		0.504		
How many servings of legumes do you consume per week? Score		0.452		
How many times per week do you consume commercial sweets or pastries (not homemade)			0.703	
How many vegetable servings do you consume per day?			0.519	
Do you preferentially consume chicken, turkey, or rabbit meat instead of veal, pork, hamburger, or sausage?			0.481	
How many servings of fish or shellfish do you consume per week?				0.686
How many times per week do you consume vegetables, pasta, rice, or other dishes seasoned with sofrito				0.599

Looking at the pattern matrix, questions about butter/margarine/cream, red meat/hamburger/meat products, and sweet/carbonated beverages loaded heavily on factor 1. Questions about nuts, fruits, olive oil use as the main culinary ingredient, and legumes heavily loaded on factor 2. Questions about commercial sweets/pastries, vegetables, and preference for chicken over meat loaded heavily on factor 3. Finally, questions about fish and homemade dishes loaded heavily on factor 4.

### Multiple correspondence analysis of 12-MEDAS items

The results of the Multiple Correspondence Analysis are shown in **Table 4**. The four dimensions accounted for 11.57% of the total inertia. As expected, the mean Cronbach’s alpha (0.305) of the four dimensions was poor. Nevertheless, a good reliability was revealed by the KR-21 (0.76).

**Table 4:** Multiple correspondence analysis of the 12-MEDAS scale items.

Dimensions from model summary	Cronbach’s Alpha	Variance Accounted For				
		Total (Eigenvalue)		Inertia		% of Variance
1	0.472	1.762		0.147		14.686
2	0.355	1.482		0.123		12.346
3	0.196	1.218		0.102		10.154
4	0.091	1.091		0.091		9.091
**Total**		**5.553**		**0.463**		
**Mean**	**0.305**	**1.388**		**0.116**		**11.569**
**Discrimination Measures**						
**12-MEDAS Items**		**Dimension**				**Mean**
		**1**	2	**3**	**4**	
Do you use olive oil as the main culinary fat?		0.002	**0.139**	0.073	0.047	0.065
How many vegetable servings do you consume per day?		0.073	0.010	**0.432**	0.000	0.129
How many fruit units (including natural fruit juices) do you consume per day?		0.006	**0.355**	0.039	0.000	0.100
How many servings of red meat, hamburger, or meat products (ham, sausage, etc.) do you consume per day?		**0.535**	0.023	0.021	0.002	0.145
How many servings of butter, margarine, or cream do you consume per day?		**0.435**	0.026	0.001	0.082	0.136
How many sweet or carbonated beverages do you drink per day?		**0.373**	0.028	0.078	0.001	0.120
How many servings of legumes do you consume per week?		**0.099**	0.094	0.020	0.073	0.071
How many servings of fish or shellfish do you consume per week?		0.008	0.050	0.129	**0.321**	0.127
How many times per week do you consume commercial sweets or pastries (not homemade)		0.124	0.175	**0.303**	0.000	0.151
How many servings of nuts (including peanuts) do you consume per week?		0.009	**0.440**	0.017	0.115	0.145
Do you preferentially consume chicken, turkey, or rabbit meat instead of veal, pork, hamburger, or sausage?		0.002	0.115	0.097	**0.163**	0.094
How many times per week do you consume vegetables, pasta, rice, or other dishes seasoned with sofrito		0.098	0.025	0.008	**0.288**	0.105
**Active Total**		**1.762**	**1.482**	**1.218**	**1.091**	**1.388**
**% of Variance**		**14.686**	**12.346**	**10.154**	**9.091**	**11.569**

### Convergent validity: Agreement between 12-MEDAS, MEDAS-FFQ, and MEDAS-24HR

**Table 5** shows the level of MD adherence according to the three tools. Weak adherence was concluded for 45.4% of the sample using the 12-MEDAS compared with 25.6% using the FFQ and 59.6% using 24HR. Moderate to fair adherence was concluded for 24.6% of the sample using the 12-MEDAS compared with 22.6% using the FFQ and 21.2% using 24HR. Good to very good adherence was concluded for 30% of the sample using the 12-MEDAS compared with 51.8% using the FFQ and 19.2% using 24HR. The correlation of the 12-MEDAS scale with FFQ/24HR is also shown in **Table 5**. Based on the Spearman rho’s correlation coefficient, the association between the 12-MEDAS and FFQ (r = 0.150) or 24HR (r = 0.240) was weak. Based on the kappa coefficient, a slight agreement was observed between the 12-MEDAS and FFQ (k = 0.027) or 24HR (k = 0.017). Based on the gamma coefficient, the association between the 12-MEDAS and FFQ (γ = 0.211) or 24HR (γ = 0.232) was weak.

**Table 5:** Correlation between 12-MEDAS adherence categories and FFQ/24HR.

**MEDAS scale categories**	**24HR**			**Total**	**FFQ**		
	**Weak Adherence**		**Moderate to Fair Adherence**	**Good to Very Good Adherence**	**Weak Adherence**	**Moderate to Fair Adherence**	**Good to Very Good Adherence**
**Weak Adherence**	159	39	29	227	69	51	107
	31.80%	7.80%	5.80%	45.40%	13.80%	10.20%	21.40%
**Moderate to Fair Adherence**	69	27	27	123	32	31	60
	13.80%	5.40%	5.40%	24.60%	6.40%	6.20%	12.00%
**Good to Very Good Adherence**	70	40	40	150	27	31	92
	14.00%	8.00%	8.00%	30%	5.40%	6.20%	18.40%
**Total**	298	106	96	500	128	113	259
	59.60%	21.20%	19.20%	100.00%	25.60%	22.60%	51.80%
**Spearman’s rho**	**r = 0.240, CI [0.153, 0.323], p < 0.001**				**r = 0.150, CI [0.06, 0.237], p < 0.001**		
**Kappa coefficient**	k = 0.017, CI [-0.034, 0.068]				k = 0.027, CI [-0.008, 0.062]		
**Gamma Coefficient**	**γ = 0.232, CI [0.089, 0.375], p = 0.002**				**γ = 0.211, CI [0.021, 0.401], p = 0.034**		

Values in **Bold** are significant. ***Abbreviations: CI*** Confidence Interval, ***FFQ*** Food Frequency Questionnaire, ***MEDAS*** Mediterranean Diet Adherence Score, ***24HR*** 24-hour Recall.

The agreement between each item of the 12-MEDAS and those of the FFQ and 24HR is shown in **Table 6**. For the FFQ items, the Kappa coefficient revealed fair agreement with the MEDAS for fruits, sweet or carbonated beverages, and nuts, and disagreement for fish or shellfish and homemade dishes seasoned with sofrito. For the 24HR, only fruits showed fair agreement with the MEDAS, and disagreement was shown for fish or shellfish and preference for chicken categories. This suggests a low convergent validity of the 12-MEDAS.

**Table 6:** Agreement Kappa coefficient between the 12-MEDAS and FFQ/24HR items.

12-MEDAS items	FFQ items	24HR items
Do you use olive oil as the main culinary fat?	k = 0.102 Slight	k = 0.032 Slight
How many vegetable servings do you consume per day?	k = 0.128 Slight	k = 0.075 Slight
How many fruit units (including natural fruit juices) do you consume per day?	k = 0.215 Fair	k = 0.212 Fair
How many servings of red meat, hamburger, or meat products (ham, sausage, etc.) do you consume per day?	k = 0.029 Slight	k = 0.026 Slight
How many servings of butter, margarine, or cream do you consume per day?	k = 0.033 Slight	k = 0.013 Slight
How many sweet or carbonated beverages do you drink per day?	k = 0.288 Fair	k = 0.114 Slight
How many servings of legumes do you consume per week?	k = 0.041 Slight	k = 0.071 Slight
How many servings of fish or shellfish do you consume per week?	k = −0.028 Disagreement	k = −0.017 Disagreement
How many times per week do you consume commercial sweets or pastries (not homemade)	k = 0.045 Slight	k = 0.122 Slight
How many servings of nuts (including peanuts) do you consume per week?	k = 0.250 Fair	k = 0.159 Slight
Do you preferentially consume chicken, turkey, or rabbit meat instead of veal, pork, hamburger, or sausage?	k = 0.094 Slight	k = −0.008 Disagreement
How many times per week do you consume vegetables, pasta, rice, or other dishes seasoned with sofrito	k = −0.015 Disagreement	k = 0.048 Slight

## Discussion

Screeners that measure and score adherence to the MD are among the most commonly used methods to assess dietary habits and evaluate their association with health outcomes. Among the various tools available, the 14-MEDAS is widely used globally and in Lebanon **[23–27]**. Despite this, the tool is not validated in Lebanon, specifically among pregnant women. The current study pioneers in presenting a comprehensive validation data of the 14-MEDAS among a sample of Lebanese pregnant women using a 157-item validated FFQ and three non-consecutive days 24HR as reference methods. Both FFQ and 24HR have been used as 14-MEDAS validation reference methods in various countries **[10–12]**.

In this study, the MEDAS screener was adapted into a 12-item scale (12-MEDAS) by removing items relevant to wine consumption and olive oil quantity. While “olive oil use” was retained, “olive oil quantity” was removed due to low communality. As part of the Lebanese culture, olive oil is rarely measured by the tablespoon; it is a “drizzled” staple, often consumed raw with *Za’atar* or *Labneh*. The screener’s focus on cooking quantity may fail to capture the significant raw intake that is culturally unique to the Levant. There is also a possibility of inadequate perception by the population of the consumed quantity, given the ubiquity of olive oil use in food preparation. Hence, the quantity of olive oil consumed may be so high across the entire population, to the point that it may lack the variance necessary to load strongly onto a specific factor, and reducing the item’s ability to discriminate between different levels of adherence within our study population. This statistical behavior highlights the necessity of cultural adaptation when applying global screeners to local contexts. From another point of view, the decision to exclude the “olive oil quantity” item while retaining other items with marginally low communalities, i.e., “legumes” and “olive oil usage”, reflects a balance between statistical rigor and conceptual validity to ensure the tool remains theoretically grounded in the original 14-MEDAS structure.

The exploratory factor analysis identified a four-factor structure, revealed a KMO value of 0.583, and an explained variance of 46.28%. While these results are borderline for sampling adequacy and construct representation, respectively, they are within the acceptable range for exploratory research in nutritional epidemiology, where dietary patterns are inherently heterogeneous. Hence, for a 12-item screener, this level of explained variance is acceptable and reflects the multifaceted nature of the MD. These results are consistent with other 14-MEDAS validation studies: for instance, the UK validation study **[28]** and the original PREDIMED validations reported similar factor structures, where items loaded onto distinct groups such as “plant-based foods”, “animal fats/proteins”, and “Mediterranean oils”. The identified factors in this study represent the diverse dietary dimensions, ranging from traditional lipid sources to vegetable intake that constitute the Mediterranean pattern in Lebanon, thereby supporting the tool’s construct validity in this population.

The MD as a complex, multidimensional construct. Accordingly, unlike reflective psychometric scales where all items are expected to be highly correlated, dietary screeners like the 12-MEDAS are formative indices, whereby the consumption of one specific food group, such as legumes, does not necessarily necessitate the high consumption of another, such as fish. Hence, the observed low Cronbach’s alpha (0.305). This value should not be interpreted as evidence of poor instrument reliability, but rather as another reflection of the formative nature of the tool, whereby a lack of inter-item correlation is expected by design. Our findings align with psychometric literature **[**29,30**]** suggesting that traditional internal consistency metrics like Cronbach’s alpha may be conceptually inappropriate for multidimensional dietary screeners, and that validity is better assessed through its correlation with reference methods and internal consistency measures suited for dichotomous items, such as KR-21.

Our findings suggest a limited agreement between the 12-MEDAS and the reference methods. These results must be interpreted within the context of the study’s methodological constraints. The low Kappa and correlation values may not solely reflect the inherent invalidity of the 12-MEDAS in Lebanon, but could also stem from limitations in the reference tools used for comparison. For instance, the FFQ and 24HR are susceptible to “shared method bias”, including significant recall and social desirability issues as well as the subjective estimation of portion sizes, potentially leading to the over- or under-reporting of certain food groups. Moreover, the use of only three 24HR, while standard, may not fully capture the high intra-individual variation in dietary intake during pregnancy. Consequently, the observed discrepancies may not solely reflect the limitations of the 12-MEDAS, but rather the cumulative errors across all three used tools. Furthermore, the retrospective derivation of the 12-MEDAS scores from FFQ/24HR data, rather than a standalone administration, and the exclusion of the wine item may have introduced additional measurement errors. Finally, in our dataset, the 12-MEDAS items are dichotomous, leading to many tied scores, which can reduce the power of the gamma statistic. Consequently, the observed “suboptimal validity” might be a conservative estimate influenced by these methodological factors. To date, the correlation between the 12-MEDAS and FFQ/24HR adherence categories remains inconsistent in the literature: while it was shown as weak in adults’ population from Bulgaria **[11]**, contrasting findings were reported in adults in Spain, Cyprus, Italy, Greece, and Portugal **[11]**. Finally, reported differences in agreement levels between our study and available literature could be attributed to the differences in assessed study populations. Our study targeted the pregnant women population, while studies done in the literature targeted different populations. The lack of studies targeting pregnant women limits comparisons, and might explain the difference in results compared with other studies, specifically in light of strong evidence showing that pregnant women have poorer general cognitive functioning and memory compared with control women **[31]**.

Regarding individual items, the highest level of agreement between the 12-MEDAS and FFQ and 24HR was observed for sweet or carbonated beverage intake (k = 0.288) and fruit consumption (k = 0.212), respectively. These findings suggest that while overall agreement levels were modest, certain food items, particularly those that are easily recalled or consumed consistently, like carbonated beverages and fruits, showed relatively better alignment across different dietary assessment methods **[32]**. This finding is inconsistent in the literature: while moderate and fair agreements were reported for fruits in Spain, Portugal, and Greece, and in Cyprus, respectively, a slight agreement was reported in Italy, and no agreement in the Republic of North Macedonia **[11]**. Other inconsistencies are noted: while a slight agreement regarding “Sofrito” was reported in our study, in Spain and Italy, findings were different for the other items **[11]**. The poor agreement for the “Sofrito” item highlights a cultural gap. While the 14-MEDAS was developed in Spain, the Lebanese culinary equivalent, stews or *“Yakhneh”*, often different vegetables, oils, and seasonings combinations. On the other hand, our study reported slight agreements for the red meat, olive oil, butter, and legumes, as noted elsewhere **[11]**. The lowest agreement in our study compared with other countries was observed for preference for chicken and fish. It could be because protein foods have lower chances of being recalled compared with other groups, such as fruits **[32]**. These correlation values are, however, consistent with existing validation literature in pregnant populations, where coefficients typically range between 0.3 and 0.5. This is potentially due to the inherent difficulty of capturing dynamic dietary intake during gestation **[12]**. Finally, the exclusion of dairy from the MEDAS is a significant limitation for pregnant women, for whom, white cheeses and yogurt, as well as *Labneh* and *Kishk* are primary calcium sources and cultural staples in Lebanon. The tool’s inability to “reward” the consumption of these healthy, culturally specific fermented dairies likely contributes to the “weak adherence” scores compared with the more detailed FFQ or 24HR.

The 12-MEDAS demonstrated limited to suboptimal relative validity in this population, suggesting that while it captures general dietary patterns, it requires cultural and physiological adaptation to accurately reflect the nutritional needs and behaviors of Lebanese pregnant women. From a research methods standpoint, the widespread use of dietary assessment tools that have not been validated in the target population raises important concerns. While tools like the 14-MEDAS have been administered in various countries, their direct adoption without local validation can compromise the accuracy of local findings. Dietary patterns, food availability, portion sizes, and cultural eating habits vary significantly across populations, and without contextual adaptation and validation, these tools may fail to accurately capture dietary intake. This can lead to misclassification, biased associations, and ultimately, less reliable public health recommendations. Researchers have the responsibility to ensure that the instruments they are using are not only evidence-based but also culturally and contextually appropriate for the populations under study **[33]**. The Lebanese Mediterranean Diet PREDIMED-derived Screener (MMPS) **[34]** looks promising in local research; however, it is important to note that it was originally validated among Lebanese women of reproductive age, rather than pregnant women. Given the physiological and dietary shifts that occur during gestation, the MMPS may serve as a useful starting point for assessment, but its diagnostic accuracy in the prenatal context remains to be formally established. Future research should focus on a head-to-head comparison between a pregnancy-adapted 12-MEDAS and the MMPS to identify which tool best captures dietary adherence to MD during pregnancy **[34]**.

Finally, the low adherence to the MD noted in this study is in line with recent literature highlighting the nutrition transition in the country **[35]**, but could be further exacerbated by the economic crisis that took place at the time of data collection: with nearly 60% of the sample earning less than USD 300, specific MD food components such as fish and nuts have become “luxury goods.”

### Strengths and limitations

This is the first in Lebanon and the region to explore the validity of the 14-MEDAS in a sample of pregnant women, against the most used tools to assess dietary patterns (FFQ and 24HR). Our findings of the suboptimal validity of this tool add to the literature an aspect that is still underexplored. We acknowledge several limitations. First, FFQ and 24HR are subject to recall issues and inaccuracies in estimating portion sizes, possibly affecting the results, specifically in pregnant women who have poorer memory **[31]**. Additionally, the reliance on self-reported data may lead to social desirability, resulting in additional bias. A main limitation of this study is its retrospective design. Because the 12-MEDAS scores were derived from previously collected FFQ and 24HR data rather than a standalone administration, we could not account for how participants might have responded to the screener as a brief, independent tool. While this method ensures the data from all three tools are synchronized in time, it may not fully capture the “real-world” performance of the MEDAS. Furthermore, this study did not assess test–retest reliability. While reproducibility is a key component of instrument validation, the rapid physiological, psychological, and behavioral shifts that occur across pregnancy trimesters, often leading to significant changes in dietary cravings and aversions, makes it challenging to establish a stable baseline for temporal reliability within this cohort. Additionally, the validation process relied on subjective dietary reference methods rather than objective biomarkers, such as serum lipid profiles or inflammatory markers. While the use of a validated FFQ and multiple dietary recalls is a standard validation approach, the lack of biological markers should be considered a limitation when interpreting the absolute accuracy of the tool in reflecting systemic nutritional status. This study is also subject to specific statistical and structural limitations. The low KMO value observed suggests that the correlations between items may not have been strong enough to support a robust factor structure, potentially limiting the depth of the EFA. Additionally, the dichotomous structure of the 12-MEDAS items, while practical for quick clinical screening, lacks the sensitivity to capture nuanced variations in dietary intake compared with continuous scales. This “all-or-nothing” scoring format may have contributed to the suboptimal validity scores by masking incremental dietary improvements or habits that fall just below the tool’s specific portion thresholds. Additionally, while efforts were made to include participants from various regions, the recruitment through healthcare settings may have introduced a selection bias. Women who regularly attend prenatal care may exhibit higher health literacy or different socioeconomic characteristics compared with those who do not have consistent access to healthcare. The final limitation of this study is the absence of a formal cross-cultural adaptation process prior to validation. Dietary habits are deeply rooted in culture, and although the 12-MEDAS covers universal Mediterranean food groups, certain nuances in portion sizes, food types, and culinary practices in Lebanon may not have been fully captured by the unmodified screener. Future research should prioritize a rigorous cultural adaptation phase to ensure that the linguistic and conceptual equivalence of the tool is optimized for the Lebanese population. Future research should also consider employing scales with higher response granularity to better capture the complexities of the Lebanese MD. It is currently impossible to compare our results with studies that used the same methodology in the same target population since they lack in the literature.

## Conclusions

While the 12-MEDAS provides a rapid assessment tool, its current form lacks the cultural and physiological sensitivity required for the Lebanese pregnant population. The discrepancy between the screener and reference methods suggests a “relative validity.” We recommend the development of a “Leb-Preg-MEDAS,” a culturally adapted version that replaces alcohol with dairy, and considers local cooking methods and eating habits reflective of Levantine stews, *Meza*, and staples, as well as use of olive oil.

## Supporting information

S1 Table Mapping of 12-item MEDAS components to FFQ and 24HR recall data. Abbreviations: g, grams; SSB, Sugar-Sweetened Beverages; Tbsp, Tablespoon.
(DOCX)

## Abbreviations

24HR: 24-hour recall
FFQ: Food Frequency Questionnaire
KMO: Kaiser-Meyer-Olkin
MD: Mediterranean Diet
MEDAS: Mediterranean Diet Adherence Screener
MMPS: Modified Mediterranean Prime Screen
