# Supplementary material for: Exploring the validity and internal consistency of the 12-point Mediterranean Diet Adherence Screener among Lebanese pregnant women
Source: PLoS One. 2026 Jun 23;21(6):e0351396. doi: 10.1371/journal.pone.0351396 (PMC13289937; doi:10.1371/journal.pone.0351396)
Supplement: S1 Table — Abbreviations: g, grams; SSB, Sugar-Sweetened Beverages; Tbsp, Tablespoon. (DOCX) [file pone.0351396.s001.docx]

**Supplementary Material**

**S1 Table.** **Mapping of 12-item MEDAS components to FFQ and 24HR recall data*.*** *Abbreviations:* ***g*** grams, ***SSB*** Sugar Sweetened Beverages, ***Tbsp*** Tablespoon

| **MEDAS Item** | **Scoring Criterion (1 Point)** | **FFQ Item Mapping** | **24HR Mapping** | **Dichotomization Threshold** |
| --- | --- | --- | --- | --- |
| **1. Olive oil use** | Main culinary fat | Olive Oil (g/d) > Other oils | Olive Oil (g/d) > Other oils | Yes (Binary: Olive oil vs. Other) |
| **2. Vegetables** | ≥2 servings/day | Q: All vegetables (excl. potatoes) | All vegetable (excl. potatoes) | ≥160g (2 servings of 80g each) |
| **3. Fruits** | ≥3 servings/day | Q: All fresh fruit | All fresh fruit | ≥312g (3 servings of 104g each) |
| **4. Red/Proc. Meat** | <1 serving/day | Q: "Beef," "Pork and charcuterie," "ham," "lamb," "Sausages" | Beef, pork, lamb, and processed meat | <100–120g/day |
| **5. Butter/Cream** | <1 serving/day | Q: "Butter," "Margarine," "Cream" | Butter and dairy cream | <12g (approx. 1 tbsp) |
| **6. Sugary Drinks** | <1 serving/day | Q: "Carbonated beverages, " "energy drink," "commercial fruit juices," "artificially flavored juices," | Carbonated and non-carbonated SSB | <250ml (1 standard cup) |
| **7. Legumes** | ≥3 servings/week | Q: "Beans," "Lentils," "Chickpeas" | All legume/pulse | ≥450g/week (cooked weight) |
| **8. Fish/Seafood** | ≥3 servings/week | Q: "Fish," "Seafood," "Tuna," "Sardines" | All fish and shellfish | ≥300–450g/week |
| **9. Commercial Sweets** | <3 times/week | Q: "Cakes," "Cookies," "Pastries" | Baked goods/dessert | <3 servings/week |
| **10. Nuts** | ≥3 servings/week | Q: "Walnuts," "pine nuts," "hazelnuts," "cashew nuts," "pistachios," "Almonds," "Peanuts" | All nut and seed | ≥90g/week (30g per serving) |
| **11. Chicken/Poultry** | Prefer over red meat | Q: Comparison of frequency | Ratio of poultry vs. red meat | Yes (if Poultry > Red Meat) |
| **12. Sofrito** | ≥2 times/week | Q: "Traditional dishes cooked with sauces" | Recipes containing oil, onion, tomato | ≥2 servings/week |
